# Risk factors, perceptions and practices associated with *Taenia solium* cysticercosis and its control in the smallholder pig production systems in Uganda: a cross-sectional survey

**DOI:** 10.1186/s12879-016-2122-x

**Published:** 2017-01-03

**Authors:** Joseph M. Kungu, Michel M. Dione, Francis Ejobi, Michael Ocaido, Delia Grace

**Affiliations:** 1 National Livestock Resources Research Institute, P. O. Box 96, Tororo, Uganda; 2 College of Veterinary Medicine Animal Resources and Biosecurity, Makerere University, P. O. Box 7062, Kampala, Uganda; 3 International Livestock Research Institute, P.O. Box 24384, Kampala, Uganda; 4 International Livestock Research Institute, P.O. Box 30709, Nairobi, Kenya

**Keywords:** *T. solium*, Risk factors, Zoonosis, Perceptions, Pigs, Humans, Uganda

## Abstract

**Background:**

Prevalence studies report *Taenia solium* cysticercosis in pig and human populations in Uganda. However, the factors influencing occurrence in smallholder pig production systems are not well documented and little is known about farmers’ perceptions of *T. solium* cysticercosis or farmer practices that could reduce transmission.

**Methods:**

To determine the risk factors, perceptions and practices regarding *T. solium* cysticercosis, a household survey using a semi-structured questionnaire was conducted in 1185 households in the rural and urban pig production systems in Masaka, Mukono and Kamuli Districts. Logistic regression was used to measure associations of risk factors with infection. Performance scores were calculated to summarise perceptions and practices of farmers regarding taeniosis, human cysticercosis and porcine cysticercosis as well as farmer behavior related to control or breaking transmission.

**Results:**

Pig breed type, farmers’ knowledge about transmission, sources of water used, and pig keeping homes where family members were unable to use the latrine were all significantly associated with *T. solium* cysticercosis in pigs. Performance scores indicated that farmers were more aware of taeniosis (63.0%; 95% Confidence Interval 60.0-65.8) than human or porcine cysticercosis; only three farmers (0.3%, 95% CI = 0.1–0.8) had knowledge on all three conditions. More farmers reported that they dewormed pigs (94.1%) than reported deworming themselves and their family members (62.0%). Albendazole was the most commonly used drug for deworming both pigs and humans (85.0 and 81.5% respectively). Just over half (54.6%) of the farmers interviewed had clean water near the latrines for washing hands. Of these, only 41.9% used water with soap to wash hands after latrine use.

**Conclusion:**

Factors that significantly influenced occurrence of *T. solium* cysticercosis in pigs were identified. Farmers had some knowledge about the disease but did not link taeniosis, human cysticercosis, and porcine cysticercosis. Therefore, there is need to employ strategies that raise awareness and interrupt transmission.

## Background

Uganda is a developing country known to be endemic for *Taenia solium* cysticercosis, a public health challenge associated with poor pig-keeping practices and sanitation. The condition has a two-stage development cycle: the intermediate (larval) and the definitive (adult) stages. The intermediate stage occurs in pigs as the primary hosts causing porcine cysticercosis and in humans as accidental hosts resulting in human cysticercosis/neurocysticercosis [1, 2]. Humans harbor the final stage, a condition called taeniosis. Cases of pigs as secondary final hosts of this tapeworm infection have been described [3]. Neurocysticercosis, a life threatening form of human cysticercosis that occurs following invasion of the brain with metacestodes, has been reported to be the chief known cause of epilepsy in human populations in pig-keeping communities in the developing countries [4]. A recent study in Zambia indicated that up to 57% of epilepsy cases reported were attributed to neurocysticercosis [5, 6]. Although no such study has been done in Uganda to estimate prevalence of human cysticercosis/neurocysticercosis, presence of *T. solium* infection in pigs is a key indicator of the occurrence of the infection in the human population. Recent serological studies in Uganda have indicated that prevalence of porcine cysticercosis in pigs ranges between 8 and 12% [7, 8].

Various factors influencing the occurrence and spatial distribution of this condition in pigs and humans have been identified [9, 10]. Such factors include: poor hygiene and sanitation practices in humans, free-range pig rearing and tethering, lack of awareness about the disease and its transmission, poor or non-inspection of pigs before or following slaughter, use of contaminated water for pigs and people, and, eating under-cooked pork [11, 12].

Specifically, poor hygiene practices such as not washing hands with soap following visits to the latrines and before eating food, eating unwashed fruits and vegetables, and, drinking un-boiled or untreated water can lead to humans ingesting the eggs of *T. solium* [13]. Poor sanitation practices, such as open air defecation and latrines in poor conditions can allow pigs access to human feces [14]. Feces deposited in the open environment can be washed into unprotected springs and wells posing a risk to both pigs and humans [12, 15].

There are 3 main pig-rearing systems practiced in Uganda: free-range, tethered, and intensive confinement where pigs are kept in corrals with or without raised floors. The extensive production systems (free-range and tethered) are usually practiced during dry season when there is a scarcity of feed and agricultural activities, which pigs might disturb, are minimal [16, 17]. Extensive systems allow pigs access to human fecal material, thereby enabling the continuity of the *T. solium* lifecycle [13]. In northern Cameroon, where the free range pig management was estimated to be 90.7%, prevalence of the condition was high (26.6%) [10]. In Zambia, it was also reported that free-range management significantly influenced occurrence of the condition [18].

The disease has been shown to be prevalent in areas where inadequate or no inspection of pork is practiced [1, 18, 19]. This is the case in most communities in Uganda where pigs are slaughtered in un-gazetted areas and uninspected pork is then sold locally or transported to urban centres for marketing [20]. This poses a serious risk to pork consumers especially when they eat undercooked pork [14].

Community awareness of a disease is crucial for control and eventual eradication. Lack of knowledge about the pork tapeworm transmission cycle by farmers, consumers and non-consumers of pork, medical and veterinary personnel, policy makers and implementers in developing countries has made control of the potentially eradicable condition difficult [21]. Limited knowledge has been linked to increasing incidence among rural poor pig-keeping communities [12, 22–24].

Stakeholders in endemic areas may know about tapeworm infections in humans but may not relate it to porcine cysticercosis and neuro-cysticercosis [12]. In Uganda, misleading reports by the media which allege that “*eating pork directly causes epilepsy*” could complicate the control of *T. solium* infection [25]. Although change of behavior in communities is not automatic after acquisition of knowledge, it could be a key step in prevention of *T. solium* cysticercosis [12, 15, 23, 24].

Given the importance of pig rearing in Uganda and the high risk that consumption of poorly cooked pork represent for the communities, this study aimed to investigate risk factors, perceptions and practices of farmers regarding taeniosis and *T. solium* cysticercosis in order to inform future control initiatives of the disease.

## Methods

### Study design

The study was conducted from April to August 2013 in 22 villages of Masaka, Mukono and Kamuli districts in Uganda. Full details of selection criteria of study sites have been reported by Ouma et al. [26]. Description of study area, sample size calculation and sampling strategy were reported by Kungu et al. [7]. In short, districts were selected as being of high potential for smallholder pig systems. Power calculation indicated a minimum sample size of 384 pigs in each district. In each village, households were randomly selected from all pig-keeping households in each village. In selected households, one pig was randomly picked and blood collected. Serum harvested was then analyzed using the HP10Ag-ELISA [27] and B158C11A10/ B60H8A4 Ag-ELISA (apDIA Cysticercosis) [28]. Every sample that tested positive in either assays contributed to the overall estimated apparent sero-prevalence of the condition.

### Household questionnaire

A questionnaire was administered to the owner of each pig that had been included in the survey to assess the risk factors for *T. solium* cysticercosis in the study sites. This questionnaire was adapted and modified from the Cysticercosis Working Group of East and Southern Africa (CWGESA) tool [29]. It was pre-tested by the first author on pig farmers in a non-study village (Mukono Municipality). It captured data on demographic characteristics, pig production and management, hygiene practices, knowledge and perceptions, as well as treatment of the condition in pigs and humans. Considering that many respondents were not fluent in English, four veterinary officers fluent in the commonly spoken indigenous language (Luganda in Masaka and Mukono, Lusoga in Kamuli) were used in each district as research assistants. Bleeding of pigs was concurrently done as already described by Kungu et al. [7]. Prior to the questionnaire administration, the study protocol was explained to the farmer and signed consent obtained.

### Statistical analysis

Data from serology, household questionnaire was entered in Microsoft excel (2010) and exported to the STATA 11 software for analysis.

#### Analysis of risk factors

Descriptive statistics for the respondents and pig characteristics were determined. A univariable analysis using logistic regression was performed to determine associations between the risk factors and sero-prevalence of *T. solium* cysticercosis (Table 1). Factors with *P*-values ≤ 0.1 were included in a model for multivariable logistic step-wise regression analysis. A backward elimination procedure was used to exclude the factors one at a time, using *P* >0.05 as the criterion. Clustering was accounted for at two levels with district as a fixed variable and village as a random effect in the multivariable models. Model diagnostics were carried out by checking for normality of residuals at village level, as well as heteroscedasticity of residuals [30]. There was minimal variation between villages considering that village level residuals were all quite small (between −1 and +1). This was also shown by the small value for the village level variance in the final model. Therefore, the fixed effects had very little effect on the size of this variance, implying that even when they were removed from the model, variation between villages still remained limited. Tests for significance of associations and odds ratios were performed at Confidence Interval of 95% and significance level of 0.05.

**Table 1:** Characteristics of respondents in the three districts

Characteristics	Category	Frequency	Percent (%)
Respondent age-group	<20 years	17	1.6
	20–40years	405	37
	41–60year	507	46.3
	>60 years	167	15.2
Sex	Female	351	32
	Male	745	68
Religion	Christian	1065	97.2
	Muslim	4	0.4
	SDA	10	0.9
	Traditional beliefs	17	1.6
Ethnic grouping	Baganda	670	61.1
	Basoga	339	30.9
	Banyankole	15	1.4
	Others	72	6.6
Level of education	Never been	114	10.4
	Primary	550	50.2
	Secondary	349	31.8
	Tertiary	83	7.6
Primary activity	Livestock	198	18.1
	Crop farming	747	68.2
	Civil service	38	3.47
	Business	59	5.38
	Others	54	4.93

#### Analysis of perceptions and practices

Performance scores were calculated for each of the five different variables used to assess knowledge on taeniosis, porcine cysticercosis, and human cysticercosis as described by Dohoo [30]. Briefly, weights of 0–10 points were subjectively assigned as overall scores to the responses on questions assessing each knowledge variable. A respondent was considered to have knowledge on a variable when his/her responses scored 8–10 points and these were then recoded into dichotomous variables (has adequate knowledge versus does not have adequate knowledge). Descriptive statistics was used to generate proportions of responses on practices associated with control of *T. solium* cysticercosis.

## Results

We sampled 375, 408, and 402 pigs in Masaka, Kamuli and Mukono, respectively. Only 1096 farmers of the 1185 whose pigs had been bled were interviewed. The remainder claimed to have commitments and left home immediately their pigs had been bled. Out of the 1185 pigs tested, 144 (12.2%) were positive by serology. Most respondents ranged from 20 to 60 years, and most were male (67.97%) and Christian by religion (97.2%). Details of the socio-demographic characteristics of the respondents are in Table 1.

### Determination of risk factors of *T. solium* cysticercosis

Several factors at the animal and household level were analyzed for their association with *T. solium* cysticercosis sero-prevalence in pigs. At animal level, six variables were assessed using univariable analysis. Only breed type had *p*-value ≤ 0.1 as indicated in Table 2. At the household level, 10 variables were assessed by univariable analysis. Level of education, knowledge of transmission cycle, water sources, and homes where people were unable to use latrine facilities had *p*-values ≤0.1 as shown in Table 3.

**Table 2:** Univariable analysis of risk factors for *T. solium* cysticercosis in pigs at animal level

Factor	Number of pigs	Seropositive pigs (%)	*p*-value	Odds (95% CI)
Pig category				
Weaner^a^	455	60(13.2)	-	-
Gilt	25	2(8)	0.457	0.572 (0.132–2.490)
Castrate	178	28(15.7)	0.406	1.229 (0.756–1.999)
Boar	177	17(9.6)	0.218	0.699 (0.396–1.236)
Sow	350	37(10.6)	0.259	0.778 (0.503–1.203)
At least grazed on pasture				
Yes	786	101(12.8)	0.611	0.899 (0.597–1.355)
No^a^	299	35(11.7)	-	-
Husbandry systems				
Intensive^a^	501	59(11.8)	-	-
Free range	13	1(7.7)	0.354	0.709 (0.342–1.469)
Tethering	577	75(13)	0.544	0.893 (0.621–1.286)
Breed type				
Local^a^	195	26(13.3)	-	-
Cross	733	104(14.2)	0.005	2.659 (1.349–5.243)
Exotic	256	14(5.5)	0.000	2.858 (1.604–5.091)
Deworm pigs				
Yes^a^	797	102(12.8)	-	-
No	388	42(10.9)	0.337	0.829 (0.566–1.215)
Source of pig				
Born on farm^a^	326	42(13.2)	-	-
Trader	758	89(11.7)	0.746	0.810 (0.227–2.898)
NGO/NAADS	15	2(12.5)	0.782	0.821 (0.119–5.675)
Gift	62	7(11.3)	0.591	0.710 (0.119–5.670)
Boar pay	19	3(15.8)	0.604	0.679 (0.157–2.930)

^a^Reference variable

**Table 3:** Univariable analysis of risk factors for *T. solium* cysticercosis at household level

Factor	Number of pigs	Seropositive pigs (%)	*p*-value	Odds (95% CI)
Level of education				
None	114	11(9.7)	0.073	0.449(0.187–1.079)
Primary	548	68(12.4)	0.06	0.452(0.197–1.033)
Secondary	348	40(11.5)	0.089	0.593(0.325–1.083)
Tertiary^a^	83	16(19.3)	-	-
Training in pig management				
Yes^a^	488	62(12.7)	-	-
No	603	73(12.1)	0.397	0.728(0.348–1.52)
Water sources				
Unprotected^a^	419	37(8.8)	-	-
Protected	677	98(14.5)	0.008	0.583(0.391–0.870)
Boil water				
Always^a^	638	79(12.4)	-	-
Never	453	56(12.4)	0.992	1.002(0.695–1.444)
Eating pork				
At least once a month	640	81(12.7)	0.281	0.653(0.3–1.418)
After a month	204	20(9.8)	0.644	0.904(0.587–1.39)
Never^a^	246	34(13.8)	-	-
Slaughter at home				
Once a year	101	8(16.5)	0.200	0.629(0.31–1.278)
After a year	21	4(19)	0.157	0.583(0.276–1.231)
Never^a^	957	123(12.9)	-	-
Inspection on slaughter				
Always^a^	11	0	-	-
Sometimes	20	3(15)	0.59	0.687(0.175–2.692)
Never	111	12(10.8)	0.999	0.000
Presence of latrine				
No	133	3(6)	0.384	0.729(0.358–1.484)
Yes^a^	1041	132(12.7)	-	-
Unable to use latrine				
Yes	595	90(15.1)	0.006	0.581(0.395–0.855)
No^a^	458	43(9.4)	-	-
Know transmission cycle				
Yes	121	26(21.5)	0.002	0.463(0.287–0.746)
No^a^	975	109(11.2)	-	-

^a^Reference variable

A multivariable logistic regression was performed to ascertain the effects of breed type, level of education, knowledge of transmission cycle, water sources, and being unable to use a latrine on the likelihood of pigs having *T. solium* cysticercosis (Table 4). Crossbred and exotic pigs were more likely to be positive than local pigs. Knowledge of the transmission cycle by farmers significantly was associated with a reduced likelihood of disease (0.476 times). Pigs from households that used water from protected sources (borehole, tap, tanks) were 0.525 times less likely to have the condition than those who used unprotected sources. Pigs in homes where all family members were able to use latrines were 0.576 times less likely to have disease.

**Table 4:** Multivariable analysis of animal and household level risk factors for *T. solium* cysticercosis

Variable	B coefficient	*P*-value	Odds ratio (95% CI)
Breed type			
Local	Reference		
Cross	1.17	0.001	3.221 (1.599–6.488)
Exotic	1.135	0.000	3.110 (1.733–5.580)
Level of education			
None	Reference		
Primary	−0.687	0.111	0.503 (0.216–1.172)
Secondary	−0.443	0.161	0.642 (0.345–1.194)
Tertiary	−0.542	0.104	0.582 (0.303–1.118)
Know transmission cycle			
Yes	−0.743	0.003	0.476 (0.291–0.779)
No	Reference		
Water source			
Unprotected	Reference		
Protected	−0.644	0.020	0.525 (0.350–0.787)
Unable to use latrine			
Yes	Reference		
No	−0.551	0.006	0.576 (0.389–0.853)

### Determination of perceptions and practices of farmers

#### Farmers’ perceptions of the three conditions

**Fig. 1:**
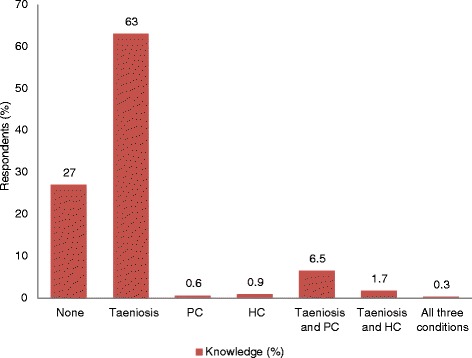
Responses (%) of farmers on knowledge of taeniosis, Porcine cysticercosis, Human cysticercosis

A knowledge performance score was conducted on the 1096 farmers’ responses related to taeniosis, human cysticercosis (HC) and porcine cysticercosis (PC) (Table 5). The proportions of the five different knowledge variables were calculated. Generally, farmers had highest knowledge on taeniosis (63.0%, 95% CI = 60.0-65.8) compared to other conditions. Only 3/1096 (0.3%, 95%CI = 0.1–0.8) respondents had knowledge about all three conditions as described in Fig. 1.

**Table 5:** Proportions of the different variables used to assess level of knowledge on the infection

Knowledge variable	Taeniosis, n (%)	Human cysticercosis, n (%)	Porcine cysticercosis, n (%)
How condition clinically manifests	782 (71.4)	56 (5.1)	319 (29.1)
How condition is acquired	780 (71.2)	22 (2.0)	127 (11.6)
Organs affected	683 (62.4)	32 (2.9)	127 (11.6)
Effects of condition	683 (62.4)	56 (5.1)	11 (1.0)
How to control condition	658 (60)	22 (2.0)	38 (3.5)

Male farmers had more knowledge about the three conditions than females. Farmers of Kamuli district, the most rural area of the study sites, had less knowledge about *T. solium* cysticercosis than Masaka and Mukono districts which are more urbanized. Table 6 shows the details of these findings.

**Table 6:** Average proportions of knowledge of the condition by gender, level of education and districts

Categories	Taeniosis (%)	Porcine cysticercosis (%)	Human cysticercosis (%)
Gender			
Male	495/745 (66.4)	107/745 (14.4)	29/745 (3.9)
Female	223/351 (63.5)	18/351 (5.1)	8/351 (2.2)
Level of education			
None	71/114 (62.3)	18/114 (15.8)	4/114 (3.5)
Primary	344/550 (62.6)	71/550 (12.9)	19/550 (3.5)
Secondary	224/349 (64.2)	18/349 (5.2)	12/349 (3.4)
Tertiary	79/83 (95.2)	18/83 (21.7)	2/83 (2.4)
District			
Kamuli	160/400 (40.0)	40/400 (10.0)	6/400 (1.5)
Masaka	259/324 (79.9)	67/324 (20.7)	16/324 (4.9)
Mukono	293/372 (78.8)	18/372 (4.8)	15/372 (4.0)

#### Control practices

Practices such as deworming of pigs and humans, as well as hand washing were assessed. More farmers reported that they dewormed pigs (94.1%) than reported deworming themselves and their family members (62.0%). Albendazole was the most commonly used drug for deworming both pigs and humans (85 and 81.5% respectively). Deworming practices varied significantly among the districts of Kamuli, Masaka and Mukono (Table 7). Just over half (54.6%) of the farmers interviewed had clean water near the latrines for washing hands. Of these, only 41.9% used water with soap to wash hands after latrine use. Availability of both water and soap varied significantly among the three districts (*X* ^2^ = 16.944, *P* < 0.05) (Table 8).

**Table 7:** Proportions of responses on deworming practices associated with control of *T. solium* cysticercosis

Deworming practice	Kamuli	Masaka	Mukono	Total, n (%)	*X* ^2^	*P*-value
Deworming pigs					4.295	0.000
Yes	303	357	371	1031 (94.1)		
No	39	18	8	65 (6.3)		
Deworm pigs how often					3.495	0.000
3 months interval	94	178	216	488 (44.5)		
Once a month	131	110	93	334 (30.5)		
> 3 months interval	84	64	61	209 (25.0)		
Drugs used						
Albendazole	122	229	209	932 (85.0)		
Ivermectin	33	23	108	164 (15.0)		
Deworming self and family					4.97	0.000
Yes	143	244	293	680 (62.0)		
No	197	129	90	416 (38.0)		
How often					2.338	0.000
Once a month	44	50	20	114 (16.8)		
3 months interval	42	117	167	326 (47.9)		
> 3 months	57	77	106	240 (35.3)		
Drugs used					2.492	0.000
Albendazole	122	228	204	554 (81.5)		
Ivermectin	0	1	0	1 (0.15)		
Praziquantel	0	0	5	5 (0.74)		
Others	23	13	84	120 (17.7)

**Table 8:** Proportions of responses on hand washing practices associated with control of taeniosis-*T. solium* cysticercosis

Practice	Masaka	Mukono	Kamuli	Total, n (%)	*X* ^2^	*P*-value
Practice hand washing					0.698	0.706
Yes	203	201	194	598 (54.6)		
No	185	163	150	498 (45.4)		
Presence of clean water and soap					16.944	0.00
Both present	172	174	113	459 (76.8)		
Only water present	217	200	220	139 (23.2)		

## Discussion

Although various factors expected to influence the transmission pattern of *T. solium* cysticercosis were assessed, only breed type, knowledge of the transmission cycle, use of water from protected sources, households with members unable to use latrines were found to be significant. We found that the odds of exotic and crossed pigs having T*. solium* cysticercosis infection were significantly higher than local ones. Similarly, Krecek et al. in South Africa reported a significantly higher sero-prevalence among crossbred pigs [31]. The pig breed types referred to here as ‘local’ have been reared for decades in the communities and are characterized by slow growth but they have adapted to the harsh conditions over time and are considered more resilient to diseases than recently introduced breeds or their crosses [32]. Also, there are some systematic differences in the way local pigs are kept and this may have influenced exposure or susceptibility. Sero-prevalence of *T. solium* cysticercosis in pigs was significantly less in homes that used protected water sources. A study in Mexico found that use of stagnant water in pigs significantly increased the prevalence in pigs [33]. Likewise, studies in Tanzania and Rwanda reported use of water from unprotected sources as an etiological factor for the condition [14, 34]. When contamination of the environment with *T. solium* eggs occurs, then the possibility for pigs and humans ingesting them is high when water from open sources such as rivers, streams, wells, and lakes is used in the homes without boiling or using decontaminating chemicals like chlorine [33]. Again confounding is possible, as households not using protected water sources are likely to differ consistently from those that do, and some of these differences could also affect pig exposure or susceptibility.

In our study, some household had latrines which some family members were not able to use. This was associated with a significant increase in porcine cysticercosis sero-prevalence. Children under age of 5 years, and weak, less mobile people (the old and sick) tend to carelessly defecate thereby increasing the risk of environmental contamination with the *T. solium* eggs. Thys et al. [12] reported that latrine use was influenced by taboos and socio-cultural beliefs thereby encouraging open-air defecation and eventual contamination of the environment with the *T. solium* eggs.

Community awareness about a disease is important for its control [35]. Our study associated knowledge of the transmission cycle by farmers with reduced likelihood of *T. solium* cysticercosis in pigs. Likewise, a study in Tanzania demonstrated that sensitization of pig keeping communities resulted in a significant reduction of the condition in pigs [21]. This study indicated that awareness of taeniosis was high among farmers compared to knowledge of human cysticercosis and porcine cysticercosis. Lack of knowledge of the latter conditions could hinder efforts of controlling the most preventable cause of epilepsy in the sub-Saharan African region [36]. Male farmers had more knowledge about the three conditions compared to female. This could be attributed to the more exposure men have at social gatherings than the women who are mostly involved in domestic work [20].

Many farmers routinely dewormed themselves and their pigs using albendazole. This practice would help limit transmission. According to the farmers, the practice was being implemented not because of their awareness about the specific dangers of *T. solium* cysticercosis but as a way of controlling worm infestations that were believed to hinder growth of the pigs and cause humans to get hungry shortly after a meal [37].

In our survey, just over half the farmers reported hand washing, similar to a nationwide survey which reports around one third of people practice hand washing (32.7%) [38]. Moreover, effectiveness of hand washing would be reduced by the low use of soap. Hand washing with soap, latrine use, and safe water use are considered by the World Health Organization as the key hygiene behaviors that limit the burden of infectious conditions like taeniosis-*T. solium* cysticercosis [39].

## Conclusion

This study indicates that a number of factors associated with etiology and persistence of *T. solium* cysticercosis exist in pig production systems in Uganda. Special considerations should be giving to making latrines accessible to children, old people and people with disabilities. Use of water from protected sources should be encouraged. Programs of sensitization about the pig tapeworm and its public health importance could raise awareness. Appropriate health education of local communities on the transmission cycle of this condition might enhance good practices such as proper hygiene and sanitation, use of water from protected sources, boiling of drinking water.

A holistic approach drawing together veterinary, medical, and public health professionals involved in activities to control taeniosis-*T. solium* conditions should be envisaged to make such efforts cheaper yet more effective.

## Abbreviation

CI: Confidence interval
